# Hyaluronic Acid-Based Nanomaterials Applied to Cancer: Where Are We Now?

**DOI:** 10.3390/pharmaceutics14102092

**Published:** 2022-09-30

**Authors:** Vera Machado, Mariana Morais, Rui Medeiros

**Affiliations:** 1 Molecular Oncology and Viral Pathology Group, Research Center of IPO Porto (CI-IPOP)/RISE@CI-IPOP (Health Research Network), Portuguese Oncology Institute of Porto (IPO Porto)/Porto Comprehensive Cancer Center (Porto.CCC), Research Center-LAB2, E Bdg 1st Floor, Rua Dr António Bernardino de Almeida, 4200-072 Porto, Portugal; 2 ICBAS, Abel Salazar Institute for the Biomedical Sciences, University of Porto, Rua Jorge Viterbo Ferreira 228, 4050-513 Porto, Portugal; 3 Biomedical Reasearch Center (CEBIMED), Faculty of Health Sciences, Fernando Pessoa University (UFP), Praça 9 de Abril 349, 4249-004 Porto, Portugal; 4 Research Department, LPCC-Portuguese League Against Cancer (NRNorte), Estr. da Circunvalação 6657, 4200-172 Porto, Portugal; 5 Faculty of Medicine, University of Porto (FMUP), Alameda Prof. Hernâni Monteiro, 4200-319 Porto, Portugal

**Keywords:** hyaluronic acid, drug carriers, drug delivery, tumor targeting, cancer

## Abstract

Cancer cells normally develop the ability to rewire or reprogram themselves to become resistant to treatments that were previously effective. Despite progress in understanding drug resistance, knowledge gaps remain regarding the underlying biological causes of drug resistance and the design of cancer treatments to overcome it. So, resistance acquisition remains a major problem in cancer treatment. Targeted therapeutics are considered the next generation of cancer therapy because they overcome many limitations of traditional treatments. Numerous tumor cells overexpress several receptors that have a high binding affinity for hyaluronic acid (HA), while they are poorly expressed in normal body cells. HA and its derivatives have the advantage of being biocompatible and biodegradable and may be conjugated with a variety of drugs and drug carriers for developing various formulations as anticancer therapies such as micelles, nanogels, and inorganic nanoparticles. Due to their stability in blood circulation and predictable delivery patterns, enhanced tumor-selective drug accumulation, and decreased toxicity to normal tissues, tumor-targeting nanomaterial-based drug delivery systems have been shown to represent an efficacious approach for the treatment of cancer. In this review, we aim to provide an overview of some in vitro and in vivo studies related to the potential of HA as a ligand to develop targeted nanovehicles for future biomedical applications in cancer treatment.

## 1. Introduction

Cancer is considered the highest clinical, social, and economic burden in terms of cause-specific disability-adjusted life years (DALYs) among all human diseases. In 2020, there were approximately 19.3 million new cancer cases and almost 10 million cancer deaths, and by 2018, in Europe alone, the total cost of cancer was EUR 199 billion [1]. For a long time, several options for cancer therapy have been developed, but successful cancer treatment remains one of the most important goals of present medical science. Current treatment approaches include surgery, chemotherapy, radiotherapy, targeted therapy, and immunotherapy. Even though they present a good cytotoxicity capacity, chemotherapy and radiotherapy lead to acute side effects (such as neuropathies, suppression of bone marrow, gastrointestinal and skin disorders, hair loss, and fatigue) and high risk of recurrences. In the case of targeted therapy, multi-drug resistance commonly occurs, limiting therapeutic efficacy, and in immunotherapy, in addition to the increased risk of autoimmune disease, a reduced efficiency against solid tumors has also been observed [2].

With the aim to enhance patients’ response to the considered anticancer treatments and to improve their general healthcare status, new advances in nanotechnology have made it possible to develop new and promising therapies based on the fundamental biology of cancer [2]. In the last several years, nanoparticles have shown great potential in numerous biomedical applications. Among them, silver nanoparticles have been studied owing to their specific physicochemical properties and their great potential in killing cancer cells [3]. Recently, it has been shown that starch-capped silver nanoparticles, synthesized through a green method, successfully induced damage in cytoplasmic membranes and mitochondria, leading to cell cycle arrest and consequent blockage of cell proliferation and death in prostate cancer cells, showing the potential of silver nanoparticles as anticancer agents [4]. Nanomedicine has been shown to overcome some of the limitations of current drugs used in cancer treatment, such as poor water solubility, lack of specificity to the tumor site, and systemic side effects [5,6,7]. In fact, several nanocarrier-based drug delivery systems have already been proposed, using materials such as liposomes, micelles, protein conjugates, and polymers, and are being tested in clinical trials [8].

Hyaluronic acid (HA) is a polymer with a much wider range of applications than the facial treatments with which it is typically associated. Recent findings and progression in research aim to demonstrate the various formulations of HA to design drug carriers and advances in HA-based drug delivery systems for promising improved cancer therapies [9]. Considering the great interest in HA from different fields and the fast-growing number of studies, a comprehensive review is needed regarding this polysaccharide and its potentialities.

## 2. Hyaluronic Acid

**Figure 1:**
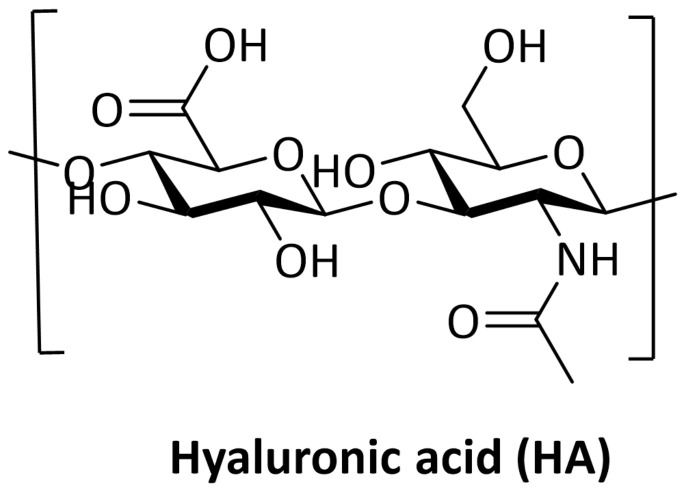
Chemical structure of Hyaluronic Acid. Created with ChemDraw Software version 12.

Hyaluronic acid is a natural anionic polysaccharide with a simple chemical structure (Figure 1) composed of two alternating repeats: disaccharide units of β-1,3-N-acetyl-D-glucosamine and β-1,4-D-glucuronic acid. It can be obtained by extraction from animal tissues, microbial production, or enzymatic synthesis. This polysaccharide is physiologically synthetized at the plasma membrane by three different hyaluronan synthases (HAS 1–3) and its molecular weight (MW) may range from 5 to 20,000 kDa in vivo [9].

It is quite difficult for the body to absorb a polysaccharide. In 2008, Nozomi Hisada and co-workers performed a study in which, using Caco-2 cells (intestinal epithelial model), they revealed that HA with a MW greater than 100 kDa is rarely absorbed. In fact, the amount of HA absorbed by Caco-2 cells increases as the MW of HA decreases to 70, 20, or 5 kDa [10]. Thus, HA is not absorbed into the body as a high-MW polymer after ingestion. The half-life of HA is very short (approximately 1–2 days in the skin and 24 h in the bloodstream). Its degradation in the human body is carried out by two distinct mechanisms: one is specific, mediated by enzymes (hyaluronidases, HYALs), while the other is non-specific, determined by oxidative damage due to reactive oxygen species (ROS). By catalyzing the hydrolysis of HA, HYALs decrease the viscosity of HA, thereby increasing tissue permeability [11].

The balance between the synthesis and degradation processes of HA plays an essential regulatory role in the human body, as it determines not only the amount of HA, but also its MW, and the MW determines the various biological actions/functions of the HA [9]. HA synthesis and degradation depends on the tissue microenvironment and is regulated by intra- and intercellular signaling factors. In cancer, the degradation of HA by HYALs is highly affected by malignancy, angiogenesis, and metastasis. The hypoxic status of a tumor and its microenvironment has a positive effect in HYALs’ activity, resulting in the production of small-sized HA fragments that promote angiogenesis and help the cancer to spread in the body. In fact, high levels of HYALs have been observed in various tumor types such as brain, bladder, and metastatic breast cancer [12]. There is no rigorous definition of high-MW and low-MW HA, but generally speaking, high-MW HA is responsible for the maintenance of the homeostatic condition, with anti-angiogenic, immunosuppressive, and anti-inflammatory properties; low-MW HA plays an opposite effect, having a key role in pathological conditions [13].

Since HA is produced by almost all cell types, in normal biological conditions, HA has multiple essential biological functions. HA can be involved in several cellular interactions (differentiation, proliferation, development, and antigen recognition) and biological functions (lubrication, hydration, matrix structure, and steric interactions). Its natural negative charge (due to the carboxylate groups) allows it to bind to a large amount of water, forming a highly viscous gel. This gel lubricates joints and acts as a buffer for the surrounding tissues, as well as contributing to tissue regeneration and remodeling processes, for example, during the healing process [14].

Owing to its high hydrophilicity, biodegradability, good biocompatibility, low toxicity, and modification flexibility, HA possesses great potential in biomedical and pharmaceutical applications, such as drug delivery systems, ophthalmic surgery, osteoarthritis treatment, and tissue engineering. It is also used in cosmetics applications, notably as dermal fillers and moisturizers [15,16]. Moreover, due to the differential expression of HA receptors in different tissues, HA also presents selectivity to target-specific sites, which increases its potential in these applications.

## 3. Hyaluronic Acid Receptors

**Figure 2:**
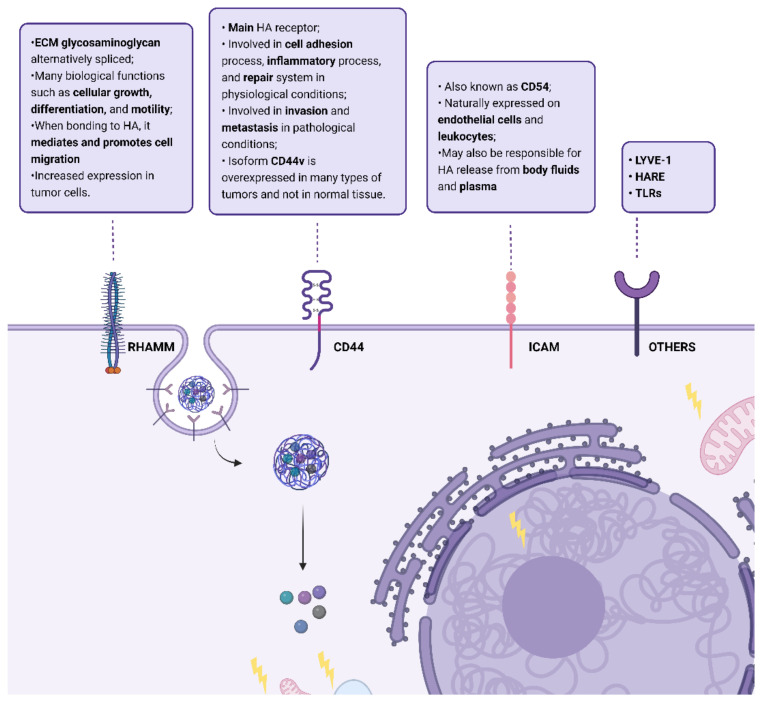
Summary of HA cell surface receptors: cluster of differentiation 44 (CD44), receptor for hyaluronic acid-mediated motility (RHAMM), Intercellular Adhesion Molecule 1 (ICAM); lymphatic vessel endothelial hyaluronan receptor (LYVE-1), hyaluronic acid receptor for endocytosis (HARE), and Toll-like receptors (TLRs) and some of their actions when bonded to HA. Created with “https://biorender.com/, accessed on 28 September 2022”.

HA is an important constituent of the extracellular matrix (ECM) that binds to ECM molecules and cell surface receptors (Figure 2), thereby regulating cellular behavior via control of the tissue’s macro- and microenvironments [11]. The three main classes of cell surface receptors for HA binding are: (1) cluster of differentiation 44 (CD44), a membrane glycoprotein, (2) receptor for hyaluronate-mediated motility (RHAMM), and (3) intercellular adhesion molecule 1 (ICAM-1).

The receptor CD44 is considered the main HA receptor and their interaction activates many pathways involved in biological processes such as inflammation, wound healing, morphogenesis, and cancer. It is endogenously expressed in different cells in normal tissues, but in low levels, and requires activation [17]. CD44 is subject to extensive alternative splicing and, thus, is a transmembrane glycoprotein family with several isoforms. In normal physiology, this receptor is involved in the cell adhesion process (aggregation and migration), inflammatory process, and repair system [18]. However, in the case of pathological physiology, as cancer, it is involved in invasion and metastasis [19]. This is due to the activation of HER2 tyrosine kinase and Src, RhoA, and Rac1, as well as to the promotion of association of CD44 isoforms to cytoskeleton proteins caused by its interaction with HA [20]. Nevertheless, in cancer cells, the structure of CD44 is modified. These cells stimulate alternative splicing and post-translational modifications, producing different isoforms of CD44 protein with enhanced binding to HA [19]. Thus, the CD44 gene can encode more than 100 isoforms, from 80 to 200 kDa. The standard isoform, CD44s, is the smaller form (85–95 kDa) without variable exons, encoded by conserved exons and is ubiquitously expressed, being composed of a single-chain molecule with various domains: N-terminal, a membrane-proximal region, comprising ligand-binding sites, a cytoplasmic domain, and transmembrane domain [16]. The isoform CD44v is the major form upregulated in cancer cells and CD44v6, a specific CD44v isoform, has been identified as the major isoform of this receptor which is overexpressed in many types of tumors, and not in normal tissue [16,21]. Additionally, CD44 has already been identified in cancer stem cells (CSCs), improving their motility, and in macrophages, making these tumors immunosuppressive [22,23].

RHAMM (also designated CD168) is an ECM glycosaminoglycan which is alternatively spliced, and its truncated forms can be found not only in the cell membrane, but also in cell cytoplasm, the nucleus, and the cytoskeleton [20,24,25]. It has a role in many biological functions such as cellular growth, differentiation, and motility. When bonded to HA, the cell surface receptor RHAMM mediates and promotes cell migration, and the intracellular RHAMM mediates the cell cycle, namely the formation and integration of the mitotic spindle [11]. This interaction is important in inflammation and tissue repair because it triggers many signaling pathways and controls cells such as fibroblasts and macrophages [26]. In the case of human cancer, it is present in solid tumors in the following organs: stomach, prostate, breast, colon, and lungs [27,28]. RHAMM is poorly expressed in the majority of common normal tissues, but shows increased expression in tumor cells, which has already been correlated with tumoral progression, invasion, metastasis development, and poor survival rate [25]. The RHAMM receptor co-exists with the CD44 receptor, which is the major cell surface HA-binding protein, but in 23% of cases, RHAMM is overexpressed in the absence of CD44 [29]. 

ICAM-1 (also known as CD54) is a cell surface metabolic receptor for HA and is naturally expressed on endothelial cells and leukocytes. Its structure is characterized by heavy glycosylation and the protein extracellular domain is composed of multiple loops created by disulfide bonds within the protein. The binding of HA to this receptor triggers a regulated cascade of events that feed the endocytic vesicles. This molecule may also be responsible for the release of HA from body fluid and plasma, which is responsible for most of its turnover throughout the body [30].

In addition to these HA receptors, others have been identified: the lymphatic vessel endothelial hyaluronan receptor (LYVE-1), the hyaluronic acid receptor for endocytosis (HARE), and Toll-like receptors (TLRs) [31].

CD44 and RHAM’s overexpression in most tumors and their correlation with poor prognosis lead to the development of therapeutic approaches through signaling targeting and drug delivery mediation by HA [16]. HA oligosaccharides (oHA) were able to abrogate signaling pathways such as the PI3K/Akt pathway and the association between CD44 and receptor tyrosine kinases. Additionally, they were able to inhibit CD44 clustering on the plasma membrane as well as block its interaction with emmprin and with different drug transporters [32]. Additionally, because of HA’s strong binding affinity for these receptors, HA has been used through targeted delivery of chemotherapeutic drugs or other novel treatments with different studies showing successful results both in vitro and in vivo [16].

## 4. Therapeutic Applications of HA in Cancer

HA represents a key molecule in a variety of medical, pharmaceutical, nutritional, and cosmetic applications since it has many useful advantages, including biocompatibility, chemical versatility, non-toxicity, biodegradability, and high hydrophilicity [31]. For many years, it has been used in the treatment of osteoarthritis, cosmetics, and in ophthalmology, but there has been a growing interest in HA’s application in other fields of medicine such as skin wound healing, tissue engineering, dentistry, and targeted drug delivery systems [13]. In recent years, HA has been studied as an anticancer delivering system, not only for drugs, but also for imaging agents, gene plasmids, and photosensitizers [16]. In fact, in the field of cancer therapy, the progress of nanotechnology facilitated the development of nanodrug delivery systems that are highly tumor-selective and allow for the slow release of active anticancer drugs, which pose as great advantages since unfunctionalized nanomaterials are potentially cytotoxic and lack cell-specific function. The concept of the “3S” transition has been recently proposed in nanotechnology referring to stability, surface, and size transition and states that if these three concepts are satisfied in drug delivery systems, all barriers in delivery processes can be overcome and the drug will be effective. HA-based nanomaterials are said to be one of the few biopolymers that can satisfy the “3S” transition approach for anticancer drugs [33]. Size is, indeed, an important factor affecting half-life in vivo and accumulation in tumor tissue. Large particles tend to stay in the tumor tissue, but their penetration ability is low, whereas small particles have the opposite characteristics, and are easily removed from blood circulation. Thus, to reach a good enhanced permeability and retention effect, drugs need to be kept in a large amount in the blood circulation and less near the tumor tissue. In the case of nanomaterials containing HA—CD44, LYVE-1, and RHAMM function as selective tumor targets. After being taken up by cancer cells through receptor-mediated endocytosis, HA is degraded to low-molecular-weight components by hyaluronidase [11]. Additionally, HA’s several functional groups (carboxylic acid, hydroxyl, and N-acetyl groups) allow several chemical conjugations and modifications and the consequent delivery of synergistic cancer therapies.

Because of these properties, HA-based nanomaterials have been studied as drug delivery systems through passive and active targeting [16]. Drug delivery systems have raised attention in overcoming drug resistance as well as increasing the therapeutic index and decreasing side effects of treatments [34]. An example of such drug delivery systems are polymeric conjugates of chemotherapy drugs. These are endocytosed, accumulating in lysosomes which leads to a release of the drug from the polymer closest to its target and makes it less prone to membrane-linked drug efflux mechanisms. Their size also constrains the extravasation of the drug to normal tissues, which diminishes toxicity. Additionally, they retain the ability to cross the irregular neo-vasculature characteristics of solid tumors and are capable of accumulating in tumor interstitium [35]. The use of nanomaterials to improve immunotherapy results has also been raising attention, since their combination can potentiate the cancer-immunity cycle through enhancement of antigen release, antigen processing, antigen presentation, and immune cell-mediated tumor killing [36]. In the same way, research regarding gene therapy has been increasing. Gene therapy delivers genetic material (such as RNA or DNA), through a vector, into the target or is used to reshape cells removed from the host which are then re-administered [37]. The use of nonviral delivery vectors, such as nanomedicine, led to lower immunogenicity and toxicity, was easier to prepare, and was able to load a higher capacity [38]. Therefore, the use of HA-based nanomaterials to deliver non-coding RNAs such as siRNAs, miRNAs, and lnc-RNAs has been studied in the last several years. Thus, the present review intends to summarize the current evidence regarding these nanomaterials and their potential application in cancer.

## 5. Evidence Acquisition

**Figure 3:**
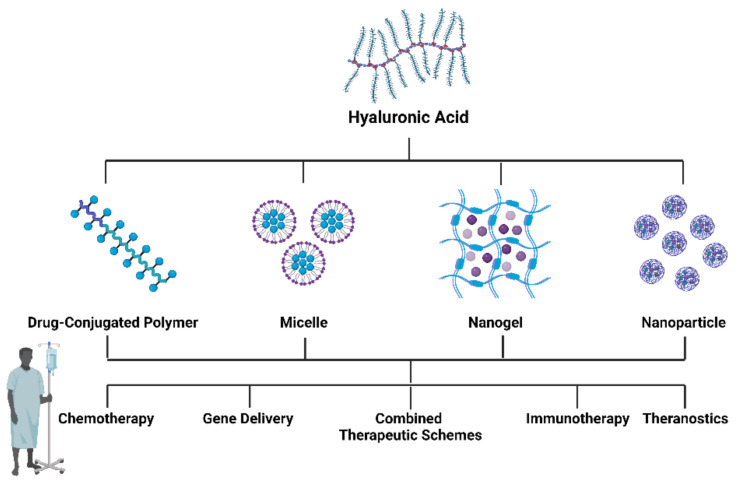
Different formulations of HA-based nanomaterials and their possible applications in cancer therapy. Created with “https://biorender.com/ accessed on 28 September 2022”.

A literature search in PubMed was conducted using the search term “Hyaluronic acid-based nanomaterials in cancer”. Papers between January 2017–March 2022 were included. A total of 366 papers were selected and after analysis and 207 papers were excluded due to the following exclusion criteria: they were review papers; we did not have access to the full text; they did not fit into the main classes of HA nanomaterials; or they were not related to cancer. The papers were then divided into major categories, corresponding to the four main classes of HA nanomaterials (Figure 3): HA–drug conjugates, HA-based hydrogels, HA micelles, and HA-based nanoparticles and their evidence was summarized in the following tables.

## 6. HA–Drug Conjugates

Taking into account the specific binding of HA to receptors on the surface of cancer cells, it can be used as a carrier of other drugs through the formation of conjugates, generating new compounds with promising antitumor effects [9]. This direct conjugation made by covalent bonds is not easily broken in the blood, but can be disrupted through hydrolysis by intracellular enzymes after reaching the target and releasing the drug [39]. Besides this targeting ability, HA–drug conjugates can improve drug solubility, stability, circulation time, and change its distribution in vivo, increasing its accumulation in tumor tissue by enhancing the osmotic retention effect. In fact, hyaluronan has already been conjugated to different antineoplastic drugs, generating new compounds with promising antitumor effects (Table 1).

Lai and collaborators have proposed the conjugation of curcumin and hyaluronic acid to form amphiphilic HA–ADH–CUR conjugates. These conjugates were efficiently internalized through CD44 receptor-mediated endocytosis by breast cancer cells and in an in vivo context. Moreover, curcumin was successfully released in an acidic lysosome environment, which is characteristic of the tumoral microenvironment, and was able to achieve significative therapeutic effects for tumor growth suppression, showing potential as a promising nanocarrier for curcumin to enhance cancer therapy with good biosafety [40]. On the other hand, DaEun Kim and collaborators conjugated S-nitrosoglutathione with HA to improve doxorubicin anticancer activity and observed that it was capable to generate NO within cells that made breast cancer cells vulnerable to doxorubicin, reinforcing its apoptotic activity. At the same time, the drug conjugate alone exhibited negligible cytotoxic effects. These results were reinforced in vivo where there was effective accumulation in the solid tumor and effective tumor growth suppression [41]. Additionally, Xiaoyu Xu and collaborators have conjugated cinnamaldehyde with hyaluronic acid and encapsulated the photosensitized protoporphyrin combining a ROS-based dual chemo/photodynamic treatment modality. The generated ROS was used as a mechanism to avoid undesired elimination of protoporphyrin and, in fact, this drug conjugate was able to induce antitumor effects both in vitro and in vivo [42].

These studies show the potential of drugs conjugated with HA as a new class of bioconjugated and tumor-targeted chemotherapeutic drugs for cancer treatment due to their innovative carrier-mediated drug delivery systems characterized by CD44-mediated endocytosis of HA and intracellular drug release with great potential.

## 7. HA-Based Hydrogel

Hydrogels are three-dimensional hydrated polymeric networks (with high water content), formed from crosslinked polymer chains with highly porous structures that enable drug release in a controlled manner [46]. In recent years, on account of their advantages such as low cytotoxicity, viscoelasticity, and bioconjugation, as well as prevention of enzymatic degradation, hydrophilic hydrogels have been widely investigated for biomedical applications such as cell therapy, tissue engineering, drug delivery, and diagnostics [47,48]. HA does not natively form physical gels alone and is susceptible to endogenous degradation; thus, the hydroxyl- and carboxyl-reactive groups in HA are often subjected to chemical modifications, crosslinking, and gelling agents to develop HA-based hydrogels with structural, mechanical, and degradation properties while maintaining native biological functions [49]. Thus, HA-based hydrogels are macroscopic networks of randomly interconnected HA chains at crosslinking points established by covalent bonds, such as hydrogen bonds, hydrophobic/hydrophilic interactions, and ionic/electrostatic interactions [50]. In the last several years, they have been studied for the controlled release of loaded anticancer drugs (Table 2).

Barbarisi M and collaborators synthesized a nanohydrogel that was able to carry quercetin combined with temozolomide and was administrated to glioblastoma cells in vitro. This nanocarrier increased the internalization of quercetin, which, when co-delivered with temozolomide, contributed to an improved anticancer effect as well as a reduction inIL-8, IL-6, and vascular endothelial growth factor (VEGF) production. The increased internalization was due to the ability of the nanohydrogel to recognize the CD44 receptor through an energy- and caveolae-dependent internalization mechanism, demonstrating the ability of hyaluronic acid nanocarriers in targeting glioblastoma cells [51]. Interestingly, Zhiwen Cao and collaborators synthesized a nanogel using hyaluronic acid and β-cyclodextrin derivative to carry auraptene and cisplatin. This nanogel showed excellent physiological stability and its delivery was affected by pH value, favoring a selective release to the tumor microenvironment. Additionally, it demonstrated a selective cytotoxicity to breast cancer cells compared to normal ones, which is a great indicator of biosafety. This is enhanced by the in vivo results, since the nanogel was able to reduce tumor volume while showing reduced systemic toxicity [52]. Nanogel application in theranostics has been demonstrated by Pan et al. [53]. They reported a one-step assembly of an HA-based multifunctional theranostic nanoplatform. Histidine was conjugated with HA and Mn2+ was used as a magnetic resonance imaging (MRI) contrast agent. Doxorubicin and chlorin e6 were then loaded as chemotherapeutic agents. This nanogel showed high biosafety and tumor microenvironment responsiveness in a melanoma cell line. The targeted responsive release of doxorubicin, chlorin e6, and Mn2+ was able to induce cell death in vitro and suppress tumor growth in vivo, showing potential both in combined chemo-photodynamic therapy and T1-weighted MR imaging [53].

Thus, the optimal formulations of hydrogels can increase the therapeutic efficacy of the local treatment of cancer, resulting in promising injectable formulations for the treatment of local and metastatic tumors.

## 8. HA Micelles

The functional groups presented in HA can be modified with hydrophobic substances such as hydrophobic drugs or polymers via esterification or amidation, allowing the binding of hydrophobic macromolecules with positive charges via electrostatic interactions to form micelles or micellar NPs for loading drugs [32]. Thus, HA can form self-assembling micelles generating amphiphilic nanocarriers. Micelles have an amphiphilic nature, displaying a spherical structure with a hydrophilic shell and hydrophobic core [62]. Therefore, they have the ability to carry hydrophobic drugs and increase their bio-availability and half-life. There are several characteristics that have made micelles the target of study in the last several years (Table 3), of which high dissolution capacity, high stability along with prolonged release, long-term circulation and the capacity to stay in the tumor for a greater amount of time are examples [63].

Tao Yu and collaborators synthesized an HA-based nanocarrier, incorporating doxorubicin and cisplatin as a CD44-targeting anticancer drug delivery system. These micelles with dual cargo were tested in breast cancer and normal cells, showing an increased drug release under acidic conditions, which is characteristic of the tumoral microenvironment. Additionally, the studies indicated a good cellular uptake and a higher cellular growth inhibition than doxorubicin and cisplatin alone. It is important to note that this was not observed in the normal breast cells, meaning there was a great polarity of the micelles to CD44+. These micelles were also tested in vivo, using a mammary cancer-bearing mouse model and, when compared to the free drugs, there was a higher inhibitory effect of the micelles, a lower toxicity, and higher tumor accumulation. These results showed the importance of HA in the formulation of nanocarriers of existing cancer drugs. [64]. On the other hand, an interesting work performed by Ying Yu and collaborators was the incorporation of a chemical radiosensitizer, doxorubicin, into the micelle’s core. These DOX-loaded ROS-sensitive nanomicelles were tested in breast cancer cells and, upon radiation stimulus, they were oxidized, generating ROS and leading to the micelles’ destruction and doxorubicin release. Additionally, when combined with radiotherapy, the DOX released by the micelles showed enhanced cytotoxicity and a sensitization of the cells to radiotherapy. This was further shown in in vivo studies in which these micelles showed longer circulation time, better tumor accumulation, and a greater tumor inhibition rate. In fact, when the tumor sites were irradiated, the release of doxorubicin was combined with the cytotoxic effect of radiotherapy with a tumor inhibition rate of about 70%. The study is an indication of the possibilities opened up by nanomedicine using HA in encapsulating anticancer drugs, maximizing their effect in combination with radiotherapy [65]. Recently, Bingjie Wang and collaborators synthesized a novel nanocarrier material for synchronous delivery of curcumin and baicalin, targeting both lung cells and tumor-associated macrophages, to effectively overcome tumor resistance. They demonstrated through in vitro cellular studies that these micelles have good cellular penetration and tumor cytotoxicity. In vivo antitumor experiments confirmed effective antitumor activity and reduced side effects in A549 tumor-bearing nude mice [66]. Even though these and other studies point to micelles as promising carriers for the delivery of anticancer drugs, there is little clinical research that proves their safety and clinical antitumor effect [78,79]. 

Another frequently used method is the coating of HA onto other nanocarriers, such as liposomes or inorganic nanoparticles, made by electrostatic attraction or covalent bonds, especially unstable bonds. A carrier system must be biocompatible, inert, and able to efficiently carry a high concentration of drug [80]. The slow release of drug from the carrier allows the drug to remain in the tumor tissue at a higher concentration and lower plasma drug concentration [81]. In Table 4, we summarize the studies with HA-based nanoparticles and their possible applications in cancer.

Carla Serri and collaborators synthesized biodegradable NPs coated with HA and loaded with gemcitabine and quercetin [82]. These HA–NPs enhanced the cellular uptake and cytotoxicity in two cell lines of pancreatic ductal adenocarcinoma, highlighting the effect of HA on targeting CD44 overexpressed in cancer cells. Furthermore, a result demonstrated the capacity of the NPs to slow the release of the incorporated drug and allow it to remain at higher concentration due to the enhancement in the anti-inflammatory properties of quercetin, showing a decrease in the interleukin cellular levels, in both cell lines pre-stimulated with lipopolysaccharides. This is an interesting result taking into account the role of interleukins in progression, metastatic processes, and drug resistance of human pancreas cancer cells, and is a study that demonstrates the benefits of using HA–NPs to improve cancer therapy [82]. Another study performed by Shaoxuan Yu and collaborators combined the advantages of curcumin, zeolitic imidazolate framework-8 nanoparticles, and hyaluronic acid for breast cancer therapy. They concluded that during storage in different media, these NPs had good stability and that under acidic conditions, a characteristic of the tumoral microenvironment, the NPs showed a slower drug release. The in vitro results obtained with these nanoparticles indicated that they have good cellular uptake which leads to several anticancer effects such as higher cytotoxicity and higher release of lactate dehydrogenase, cell cycle arrest, induction of apoptosis and production of reactive oxygen species. In this study, in vivo experiments were also performed, using mammary cancer-bearing mice models, showing that these NPs are able to strongly inhibit the tumor growth and pulmonary metastasis, remarking the properties obtained with the introduction of HA in the nanoparticles [83].

Taking into account that combinational cancer therapy has been considered a promising strategy to achieve synergetic therapeutic effects and suppression of multidrug resistance, in 2018, Yang Li and collaborators developed a dual-targeting delivery system [163]. These NPs were based on a ligand of CD44 receptors (1,2-distearoyl-sn-glycero-3-phosphoethanolamine-hyaluronic acid) and a selective ligand of folate receptors (MTX) with a focus on combining methotrexate (MTX), which act on cytoplasm, and 10-hydroxycamptothecin (HCPT), an alkaloid acting on nuclei, to treat breast cancer. The efficiency of selective internalization of these NPs via CD44/folate receptors was confirmed by cellular uptake results. Additionally, in vivo studies indicated NP accumulation at the tumor sites through passive-plus-active targeting, leading to synergetic tumor cell death and inhibition of tumor growth, showing that these NPs can be an efficient delivery system for tumor-targeting therapy [163]. Another example of a dual-targeting delivery system was performed in 2019 by Safia Naz and collaborators based on mesoporous silica NPs, which performed as a drug delivery system, demonstrating enzyme-responsive and multistage-targeted anticancer effects with doxorubicin (DOX) as a cargo. To obtain this delivery system, the authors grafted the mesoporous silica NPs with triphenylphosphine (TPP) and capped them with HA. The resulting NPs had dual-targeting, CD44 (HA), and mitochondrial-targeting properties (TPP), which was confirmed by the results showing that cancer cells favorably uptake these NPs via CD44 receptor-mediated endocytosis and are largely accumulated in mitochondria. In cancer cells, overexpressed HAase enabled HA degradation leading to the enzyme-responsive release of DOX, killing cells while exhibiting much lower cytotoxicity than normal ones [84].

Additionally, Mengjiao Zhou and collaborators synthesized carrier-free drug NPs that carry paclitaxel and DOX modified with cis-aconitic anhydride, coated with a crosslinker based on HA. Based on the unique pH and redox environment of tumor tissues, the objective of the authors was to obtain NPs with pH- and redox-responsive release capable of CD44 targeting. The results showed that these NPs at a neutral pH level, such as that of the blood stream, are stable and have a very low drug release. However, at acidic pH levels, such as that in the tumor environment, they observed a significant increase in drug release. The authors also tested the tumor selectivity, using normal and cancer cells, concluding that these NPs preferentially target cancer cells, expected, due to the presence of HA. Further, In vivo studies showed high accumulation in tumors and excellent inhibition of tumor growth. The authors observed a greater anticancer effect than the individual paclitaxel and DOX; together, these results presented the ability of HA–NPs in targeting cancer cells and increasing the drug availability [85]. In 2018, Dalia Kabary and collaborators, focusing on lung cancer therapy, developed inhalable nanocomposites with the ability to deliver the hydrophobic mTOR inhibitor rapamycin (RAP) and the hydrophilic herbal drug berberine (BER) [148]. In order to decrease the delivery gap between the two drugs, the authors created two types of multi-compartmental nanocarriers by enveloping a BER hydrophobic ion pair-lipid nanocore within a shell of RAP-phospholipid complex bilayer. Then, they were coated with cationic lactoferrin and anionic hyaluronate to improve their tumor targeting. The authors performed in vivo studies using lung cancer-bearing mice, in order to compare the anticancer efficiency of inhaled free drugs to the inhalable nanocomposites, and it was possible to see a remarkable decrease in lung weight and in the number and diameters of lung adenomatous foci and angiogenic markers. This study showed a potential application of NPs for localized delivery to tumor cells via inhalable multi-compartmental nanocomposites, which is promising in the management of lung cancer [148]. In 2020, Jinying Liang and collaborators created and characterized lipid/hyaluronic acid (HA)-coated DOX–Fe_3_O_4_ and determined its safety and effectiveness on breast cancer [98]. As it was described, DOX was conjugated onto the Fe_3_O_4_ NP surface and then coated with a tumor cell-targeting HA ligand, phosphatidylcholine (PC) lipid, in order to obtain a dual-targeting NP. The objective of the authors was to obtain a drug delivery system capable of transporting DOX into cancer cells, decreasing its cardiotoxicity and addressing any MDR problems. The results showed the synergistic interaction of this coated PC/HA surface with DOX–Fe_3_O_4_, resulting in good antitumor efficacy for MDR cancer therapy and diminutive DOX cardiotoxicity, showing the potential of PC/HA@DOX–Fe_3_O_4_ NPs as efficient nanocarriers to overcome MDR tumors and the cardiotoxicity of DOX [98]. In the same year, Jing Yang and collaborators used HA as an alternative to plasmid DNA to construct a novel type of cationic liposome carrier that can carry siRNA [192]. The objective of the investigators was to create a carrier that targets melanoma survivin and evaluate the efficacy of this carrier and the potential of this target. They concluded that these NPs inhibit melanoma proliferation in time and dose matters, in vitro, and target survivin. In addition, NPs inhibited the metastatic ability of melanoma cells. In the in vivo experiments, the cationic liposome NPs were injected into a mouse tumor model, and an inhibition of tumor growth and a significant reduction of the expression of survivin mRNA and protein were observed. This is a good study that shows that siRNA cationic liposome NPs are highly stable and have notable properties of low immunogenicity and toxicity, and can effectively inhibit melanoma cells by inhibiting survivin expression [192].

Together, the studies discussed here and the ones presented in the Table 4 show that HA–NPs are a highly promising nanocarrier and are efficient as a delivery system to perform enhanced cancer therapy with good biosafety.

## 9. Conclusions and Future Perspectives

Considering the numerous publications in recent years, it is clear that nanomaterials with hyaluronic acid as a biomaterial designed to target different tumors are perceived as a promising and attractive strategy to improve cancer therapy.

The application of nanomaterials in the field of biomedicines has had a great impact on the delivery of anti-neoplastics. In the last several years, the development of nanodrug delivery systems for targeted tumor treatment has been the focus of many researchers, namely regarding glycosaminoglycans that target CD44, since this receptor is overexpressed in different tumor cells. HA-based nanomaterials have potential applications in chemotherapy, gene therapy, immunotherapy, and combination therapy for cancer treatment due to the negatively charged surfaces that make them beneficial for prolonged blood circulation, protecting drugs from absorption by endothelial cells. Additionally, all biological properties of HA—such as the cell surface receptors with which HA can interact, including the CD44 receptor, which is widely expressed in cancer cells when compared to normal ones—are a relevant aspect. It is the interaction with these receptors that enables targeted delivery to target locations, resulting in greater cellular uptake and, therefore, beneficial results.

Nevertheless, there are some major points of potential improvement in order to overcome some of the possible obstacles that make it difficult to translate HA-based nanomaterials to clinical applications. Firstly, more extensive and in-depth investigation needs to be performed in order to improve the uptake of the different biomaterials, namely regarding CD44 binding. Secondly, some chemical modifications in the HA structure may affect CD44 targeting and also affect HA degradation, causing undesirable cellular uptake and drug release. Thus, the study of drug release and nanomaterial degradation kinetics is highly needed to improve the ability to apply these biomaterials in the clinical practice. One of the main concerns regarding hyaluronic acid’s ability to target the nanomaterial to tumor cells is that CD44 is expressed in normal cells, even if in a lower concentration. Therefore, the possibility to improve these nanomaterials would involve modifying it in order to target CD44v, an isoform of CD44 expressed in tumor cells. The path to clinical implementation of a drug is long, and both 3D spheroids and in vivo studies are needed to strengthen the potential of these nanomaterials and their biosafety in order to apply them both as therapeutic agents and also as theranostic agents. More specifically, there is a lack of studies regarding biodistribution, toxicity, and availability in physiological conditions. The study of these parameters is strongly needed in order to clinically implement hyaluronic acid-based nanomaterials.

## Figures and Tables

**Table 1:** Recent application of HA-based drug conjugates in cancer models.

Compound/Drug	Status	Model	Effect	Role of Nanomaterial	Ref.
Curcumin	In vitro In vivo	Breast cancer	Efficiently accumulates in tumor site via EPR effect and CD44-mediated endocytosis; Antitumor effect.	Nanocarrier	[40]
Doxorubicin	In vitro In vivo	Breast cancer	Efficient delivery into cancer cells; Increases the therapeutic and the apoptotic activity of DOX; Effectively suppress tumor growth in vivo.	Chemosensitizing agent	[41]
Cinnamaldehyde and protoporphyrin	In vitro In vivo	Melanoma	Improves bioavailability and selective tumor accumulation; Induces cytotoxic ROS generation; Improves antitumor performance.	Delivery system and photodynamic therapy	[42]
Doxorubicin	In vitro In vivo	Hepatocellular carcinoma	Excellent antitumor capability.	Drug delivery system	[43]
Doxorubicin	In vitro	Cervical cancer	Much better cellular uptake and higher cytotoxicity in tumor cells than normal ones.	Drug delivery system	[44]
siRNA	In vitro In vivo	Glioblastoma	Efficiently delivers into tumor cells/tissues and mediates less cytotoxicities in normal cells; Significantly enhances antitumor ability.	siRNA delivery	[45]

**Table 2:** Recent application of HA-based hydrogels in cancer models.

Compound/Drug	Status	Model	Effect	Role of Nanomaterial	Ref.
Quercetin combined with Temozolomide	In vitro	Brain cancer (Glioblastoma multiform)	Proficient in mediating site-specific delivery of quercetin via CD44 receptor; Improves the therapeutic efficacy of temozolomide by modulating brain tumor microenvironment.	Drug delivery system	[51]
Auraptene and Cisplatin	In vitro In vivo	Breast cancer	Excellent physiological stability and fluorescence effects; Selective internalization; Antitumor effects and lower systemic toxicity.	Dual-targeted delivery and synergistic therapy	[52]
Doxorubicin	In vitro In vivo	Melanoma	High biosafety; Tumor microenvironment responsiveness; Ability to target CD44 overexpressed in melanoma cells; Ability to suppress tumor growth in vivo.	Drug delivery system	[53]
Oncolytic viruses	In vitro	Colorectal Cancer Prostate Cancer	In vitro cytotoxicity assays demonstrate good oncolytic activity of OV-loaded nanohydrogel against cells.	Delivery system	[54]
Coumarin	In vitro In vivo	Cervical Cancer	The results provide novel insights into several aspects of the in vitro and in vivo behavior of nanogels.	Drug delivery system	[55]
EF2-Kinase inhibitor	In vitro	Breast cancer Pancreatic cancer	Inhibition of cell proliferation and colony formation of breast and pancreatic cancer cells.	Drug delivery system	[56]
Quercetin and Everolimus	In vitro	Breast cancer	Synergistic cytotoxic effects; Antitumor and anti-inflammatory properties.	Nanocarrier	[57]
Polypyrrole and doxorubicin	In vitro In vivo	Breast cancer	Significant inhibition of a subcutaneous tumor model through combined photothermo-chemotherapy under laser irradiation.	Drug delivery system	[58]
Paclitaxel and interferon gamma	In vitro	Lung carcinoma	Positive effects on cancer cells and fewer side effects on healthy ones.	Drug delivery system	[59]
Doxorubicin	In vitro	Hepatocellular carcinoma	Excellent DOX-loading capacity; Cytotoxicity induction.	Drug delivery system	[60]
C14-Gemcitabine	In vitro	Colon and Pancreatic cancer	Controlled release of drug; Potential for intratumoral delivery of anticancer agents.	Drug delivery system	[61]

**Table 3:** Recent application of HA-based micelles in cancer models.

Compound/Drug	Status	Model	Effect	Role of Nanomaterial	Ref.
Doxorubicin and Cisplatin	In vitro In vivo	Breast Cancer	Enhanced drug release under acidic conditions and higher cellular uptake; Stronger cellular growth inhibition and lower systemic toxicity than free drugs.	Drug delivery systems	[64]
Doxorubicin	In vitro In vivo	Breast cancer	Combined with radiotherapy, ROS-sensitive micelles disintegrated and released great drug cargos, enhancing cytotoxicity; Prolonged circulation time and improved tumor accumulation.	ROS-sensitive drug delivery system	[65]
Curcumin and Baicalin	In vitro In vivo	Lung cancer	Good cellular penetration and tumor cytotoxicity; Effective antitumor activity and reduced side effects.	Drug delivery system	[66]
Vitamin E Paclitaxel	In vitro In vivo	Breast Cancer Melanoma	Strong antineoplastic effects due to redox responsiveness; Excellent tumor-targeting ability and prolonged retention time compared to Taxol in vivo.	Drug delivery system	[67]
Cisplatin	In vitro In vivo	Ovarian cancer	Prolonged blood circulation and preferential tumor accumulation; higher antitumor efficacy.	Drug delivery system	[68]
Gambogic acid	In vitro In vivo	Lung cancer	Higher apoptosis induction and cytotoxicity.	Drug delivery system	[69]
Lauroyl-gemcitabine and honokiol	In vitro In vivo	Glioblastoma multiforme	Stronger inhibition of glioma proliferation and apoptosis induction.	Delivery system	[70]
Doxorubicin	In vitro	Cervical cancer	Nanomicelles could be disassembled upon UV light; Inhibition of proliferation.	Drug delivery system	[71]
Tocopherol succinate	In vitro In vivo	Melanoma	Greater tumor accumulation; Higher antineoplastic responses.	Drug delivery system	[72]
Indocyanine green derivative and paclitaxel	In vitro In vivo	Breast cancer	Improved stability and reduced systemic toxicity; High stability, smart release behavior, and excellent tumor-targeting ability; Great synergy in tumor inhibition.	Delivery system	[73]
Tirapazamine	In vitro In vivo	Breast cancer	Efficient activation of mitochondrial apoptosis cascade and oxygen depletion in the tumor intracellular environment to amplify the hypoxia-dependent cytotoxic effect of TPZ.	Delivery system	[74]
Tamoxifen	In vitro Ex vivo	Breast cancer	Safe and compatible against macrophages; Efficiently kills cancer cells; non-toxic nature in contrast to pure TMX; Augmented intracellular uptake with strong targeting potential for anti-proliferative activity.	Drug delivery system	[75]
Oxygen	In vitro In vivo	Ocular choroidal melanoma	Increased generation of O_2_ and elevated phototoxicity.	Delivery system	[76]
Doxorubicin	In vivo	Breast cancer	Remarkable therapeutic effect and minimized toxicity in vivo.	Light-activated drug release	[77]

**Table 4:** Recent application of HA-based nanoparticles in cancer models.

Compound/Drug	Status	Model	Effect	Role of Nanomaterial	Ref.
Gemcitabine and Quercetin	In vitro	Pancreatic ductal adenocarcinoma	Improved cytotoxicity and cellular uptake; Improved anti-inflammatory properties of quercetin and decrease in interleukin cellular levels.	Drug delivery system	[82]
Curcumin	In vitro In vivo	Breast cancer	Cellular uptake and higher cytotoxicity; Higher lactate dehydrogenase release, cell cycle arrest in G2/M, S phases, ROS generation, and apoptosis; Stronger inhibitory effect on tumor growth and pulmonary metastasis.	Drug delivery system	[83]
Doxorubicin	In vitro	Gastric cancer	Preferentially taken up by cancer cells; Mainly accumulated in mitochondria; Efficiently killed cancer cells.	Drug delivery system	[84]
Doxorubicin and paclitaxel	In vitro In vivo	Lung and Breast cancer	High stability, excellent active targeting effect and controllable intracellular drug release and, ultimately, better anticancer efficiency than individual drugs.	Co-delivery system	[85]
Docetaxel	In vitro In vivo	Breast cancer	Antitumor effect.	Drug delivery system	[86]
Pentamidine isethionate	In vitro	Lung Adenocarcinoma Breast cancer	More cytotoxic in comparison to the free drug, suggesting an enhanced internalization of encapsulated drug by cancer cells.	Drug delivery system	[87]
Hyaluronic acid-ceramide	In vitro In vivo	Breast cancer	Additional tumor-targeting and penetration potential together with enhanced permeability and retention (EPR) effect (passive tumor targeting) and HA–CD44 receptor interaction (active tumor targeting).	Nanocarrier for imaging and therapy	[88]
IR780Doxorubicin	In vitro	Breast cancer	Increased photothermal potential and cytocompatibility of IR780; Higher internalization by cancer cells than by normal ones; Decrease in spheroid cell viability.	Cancer chemo-phototherapy Co-delivery system	[89]
Catalase	In vitro In vivo	Breast cancer	Minimal cytotoxicity in the dark and high toxicity under 660 nm light irradiation at normoxic conditions; Selective tumor accumulation in tumor-bearing nude mice; Significant tumor regression after intravenous injection under light irradiation compared to control system without loading catalase.	Photodynamic therapy	[90]
Doxorubicin	In vitro In vivo	Lung Adenocarcinoma	Antitumor effects and minimal systemic toxicity.	Nanocarrier	[91]
Curcumin	In vitro In vivo	Breast cancer	Cell death by ROS induction, cell cycle arrest, and modulation of NF-κB and Bax-mediated apoptotic pathway; Decreased tumor volume in tumor-bearing mice due to increased bioavailability and higher cellular uptake in tumor tissue.	Drug delivery system	[92]
Doxorubicin and cisplatin	In vitro	Breast cancer	DOX and cisplatin exhibited a synergistic cell-killing effect in human breast cancer MCF-7 cells.	Synergetic targeted combination chemotherapy	[93]
Doxorubicin	In vitro	Breast Cancer	Excellent targeting of cancer cells.	Drug delivery system	[94]
Cisplatin	In vitro In vivo	Human ovarian cancer; Ehrlich tumor (solid)-bearing mice	Higher cytotoxicity than the free drug; in vivo antitumor activity.	Drug delivery system	[95]
Lapatinib	In vitro In vivo	Breast cancer	Improved antiproliferation potential, apoptotic efficacy, and mitochondrial destabilizing activity; tumor growth suppression.	Drug delivery system	[96]
Paclitaxel	In vitro In vivo	Colorectal and Breast cancer; Lung adenocarcinoma; Hepatocellular carcinoma; Melanoma	Effective tumor ablation with minimal adverse events; Significantly inhibited melanoma tumor growth.	Drug delivery system	[97]
Doxorubicin	In vitro In vivo	Breast cancer	Greater cellular uptake and cytotoxicity; Significant tumor-targeting capabilities and tumor growth inhibition activity with less cardiotoxicity.	Drug delivery system	[98]
IRDye800CW Camptothecin	In vitro In vivo	Breast cancer	High-precision tumor therapy with no tumor recurrence and metastasis.	Drug delivery system Chemo-photothermal therapy	[99]
Zinc(II) phthalocyanine-based photosensitizer	In vitro In vivo	Colorectal adenocarcinoma; Lung adenocarcinoma	Upon irradiation, NPs caused significant temperature increase at the tumor site and ablation of the tumor. Effective photothermal agent for targeted photothermal therapy.	Nanocarrier for photothermal therapy	[100]
Thio-tetrazolyl analog of a clinical candidate, IC87114	In vitro	Pancreatic cancer Breast Cancer	Higher cytotoxicity and enhanced intracellular accumulation of NPs in high-CD44-expressing cells; Induction of premature senescence with increase in senescence-associated β-galactosidase activity and senescence-specific marker p21 expression through modulation of Pi3K/Akt/NF-kB.	Nanocarrier	[101]
Doxorubicin	In vitro	Cervical cancer	Higher cellular uptake via CD44 receptor-mediated endocytosis and higher cytotoxicity in Hela cells compared to normal ones.	Drug delivery system	[102]
Horseradish peroxidase or indole-3-acetic acid	In vitro	Bladder cancer	Reduction of the cell viability of human bladder carcinoma cell line.	Delivery of enzyme/prodrug systems	[103]
Gefitinib and Vorinostat	In vitro In vivo	Lung cancer (2D and 3D cultures)	Stronger inhibition of orthotopic lung tumor growth compared to free drugs.	Co-delivery system	[104]
Zinc oxideGinsenoside Rh2	In vitro	Lung and Colorectal adenocarcinoma; Breast cancer	Induction of apoptosis through generation of ROS by activation of the Caspase-9/p38 MAPK pathway.	Drug delivery system	[105]
Curcuminoid	In vitro In vivo	Malignant glioma	Effectively targeted and accumulated within the gliomas after enhanced permeation through blood–brain barrier.	Drug delivery system	[106]
Olaparib	In vitro In vivo	Triple-negative breast cancer	Antitumor effect.	Drug delivery system	[107]
Honokiol	In vitro In vivo	Breast cancer	Improved antiproliferative and proapoptotic activities; Downregulation of the expressions of Vimentin and upregulation of E-cadherin.	Drug delivery system	[108]
TRAIL plasmid and gambogic acid	In vitro In vivo	Breast cancer	Significantly augmented apoptotic cell death; inhibited TNBC tumor growth; efficiently co-delivered GA and pTRAIL.	Co-delivery system	[109]
Doxorubicin	In vitro In vivo	Breast cancer	Improved the cellular uptake and cytotoxicity; Inhibited tumor growth.	Drug delivery system	[110]
Doxorubicin	In vitro In vivo	Breast cancer	Specific uptake by the tumor; Better therapeutic efficacy.	Drug delivery system	[111]
Diaminocyclohexane-platinum	In vitro In vivo	Lung cancer	Anticancer activity; Ability to modulate immunogenic cell death.	Drug delivery system	[112]
Docetaxel	In vitro In vivo	Lung cancer	Fast cellular uptake; Improved tumor accumulation and repression and lower side effects compared with free docetaxel.	Drug delivery system	[113]
Doxorubicin, cisplatin and resiquimod	In vivo	Osteosarcoma	The growth of tumors and lung metastasis was greatly inhibited.	Intelligent co-delivery platform	[114]
Doxorubicin	In vitro In vivo	Breast cancer	Mitochondrial destruction and nuclear DNA leakage led to cell cycle arrest and cell apoptosis; Effective tumor inhibition.	Drug delivery system	[115]
Doxorubicin	In vitro In vivo	Colorectal cancer	Significantly increased DOX circulation time by 12.5 times; Efficiently targeted tumor tissues; Antitumor effect.	Drug delivery system	[116]
Camptothecin	In vitro	Lung cancer	Recognizes normal cells and cancer cells and has good anticancer effects.	Drug delivery system	[117]
Doxorubicin	In vitro In vivo	Breast Cancer Brain Metastases	Selective cytotoxicity to metastatic breast cancer cells rather than astrocytes; Efficient loading into dual-targeting NPs; Significantly extended the median survival time of mice with intracranial metastatic breast cancer.	Delivery system	[118]
OligoRNA and Doxorubicin	In vitro In vivo	Hepatocellular carcinoma	Effective delivery of doxorubicin and oligoRNA into cells via CD44 receptor-mediated endocytosis; Significantly inhibited cell proliferation; Efficient accumulation in tumor.	Co-delivery system	[119]
Gambogic acid	In vitro	Melanoma	Improved cytotoxicity; Induced apoptosis and mitochondrial depolarization; Inhibited tumor metastasis.	Drug delivery system	[120]
Berberine and Doxorubicin	In vitro In vivo	Hepatocellular carcinoma	Enhanced antitumor activity, tumor accumulation, and biocompatibility.	Co-delivery system	[121]
Paclitaxel	In vitro	Breast cancer	Improved cellular uptake.	Drug delivery system	[122]
Photosensitive drug indocyanine green	In vitro In vivo	Lung cancer	Excellent drug loading and stability; Significant uptake.	Photothermal/photodynamic therapy	[123]
Dopamine	In vitro In vivo	Breast cancer	Enhanced cellular accumulation efficiency, antiproliferation property, tumor penetration efficiency, and spheroid growth inhibitory effect.	Tumor-targetable and penetrable nano-system	[124]
Doxorubicin and photothermal reagent indocyanine green	In vitro In vivo	Cervical cancer	Improved effectiveness of photothermal therapy; Excellent synergistic therapy.	Bimodal imaging	[125]
Doxorubicin	In vitro In vivo	Liver cancer	Prolonged drug blood circulation time; Increased accumulation of drug in the liver and decreased cardiotoxicity and nephrotoxicity; Tumor targeting.	Drug delivery system	[126]
Mitoxantrone	In vitro	Breast cancer	Specifically bound to and significantly inhibited CD44 receptor-positive cells.	Drug delivery system	[127]
Doxorubicin	In vitro	Cervical cancer	Higher tumor cell inhibition ratio; Efficient cellular uptake.	Drug delivery system	[128]
Paclitaxel	In vitro In vivo	Breast cancer	Anticancer efficacy; NPs accumulated in tumor site; Enhanced apoptosis; Reduced tumor growth.	Drug delivery system	[129]
Docetaxel and Disulfonate Tetraphenyl Chlorin	In vitro	Breast cancer Cervical cancer	Synergistic drug/treatment interaction; Induced cell mortality.	Co-delivery system	[130]
Curcumin and 5-fluorouracil	In vitro In vivo	Breast cancer	Synergistic anticancer, proapoptotic, and anti-migration effects; Anticancer activity against metastatic breast cancer.	Co-delivery system	[131]
Berberine chloride	In vitro In vivo	Cervical and breast cancer Ehrlich Ascites Carcinoma	Faster release of BRB and increased cytotoxicity; Enhanced apoptosis, sub-G1 content, life span, mean survival time, and ROS levels with subsequent decrease in mitochondrial membrane potential and tumor burden.	Delivery system	[132]
Triptolide	In vitro In vivo	Breast cancer	High drug loading efficiency; Selective tumor cellular uptake and high tumor tissue accumulation capacity; Suppression of cell proliferation; Blockage of proapoptotic and cell cycle activities; Strong inhibition of cell migration and invasion.	Drug delivery system	[133]
Doxorubicin and Ce6	In vitro In vivo	Lung carcinoma	Tumor site-specific light irradiation generated high levels of ROS and greatly enhanced the hypoxic levels to induce NP dissociation and drug release. A synergistic anticancer efficacy and reduced side effects to normal cells.	Co-delivery system	[134]
Tirapazamine and Ce6	In vitro In vivo	Breast cancer	Effective tumor accumulation; High levels of ROS.	Drug delivery system (photodynamic therapy)	[135]
Dissolving microneedles and photothermal agent (CuS)	In vitro In vivo	Melanoma	Improved specific uptake and distribution of targeted tumor; Delivers drug locally; Releases drug intelligently and spatiotemporally.	Co-delivery system	[136]
Paclitaxel and lethal-7a (let-7a), a microRNA (miR)	In vitro In vivo	Ovarian cancer	Effective cellular uptake; Significant downregulation of P-glycoprotein; Efficient drug release and induction of apoptosis; Synergistic growth inhibition.	Co-delivery system	[137]
Camptothecin	In vitro	Lung cancer	Easily taken up by mitochondria; Severe mitochondrial dysfunction; Rising cell death rate.	Drug delivery system	[138]
Doxorubicin	In vitro	Breast and Liver cancers	Exhibited an endosomal escape function to accelerate drug release in cancer cells, leading to low IC50.	Drug delivery system	[139]
Melittin and condensed epigallocatechin gallate	In vitro In vivo	Melanoma	Synergistic amplification of oxidative stress and prolonged ROS retention in cancer cells; Enhanced anticancer efficacy.	Drug delivery system	[140]
5-Amino levulinic acid and artemisinin	In vitro In vivo	Hepatoma	Tumor targeting; antitumor effect; Good multi-functional therapeutic delivery system.	Co-delivery system	[141]
All-trans-retinoic acid	In vitro In vivo	Lung cancer	Tumor growth inhibition; Efficient system for targeted delivery of antitumor drugs to eliminate cancer stem cells.	Drug delivery system	[142]
Doxorubicin and a near-infrared dye (indocyanine green)	In vitro In vivo	Breast cancer	Fluorescence imaging ability and release of the drug; Generation of high heat upon NIR irradiation and induction of apoptosis; Inhibition of tumor growth with minimal systemic toxicity upon NIR irradiation.	Multifunctional drug delivery system for cancer therapy and imaging	[143]
Gambogic acid	In vitro In vivo	Hepatocellular carcinoma	Induction of reduction-activated charge conversion from about -25 to +30 mV with up to 95% drug release within 48 h; Excellent tumor inhibition.	Delivery system	[144]
Antitumor immune regulator (R848) and Doxorubicin	In vitro In vivo	Immune cells and Breast cancer	Strong immunoregulatory activities; Inhibited the breast cancer cell growth; Excellent tumor-targeting ability and inhibition of tumor growth by regulation of tumor immunity.	Co-delivery system	[145]
Cisplatin–indocyanine green	In vitro In vivo	Hepatocellular carcinoma	Ultra-high drug loading efficiency and glutathione/NIR light dual-responsive drug release; Efficient internalization and apoptosis-inducing ability; Efficient tumor accumulation, biosafety, and synergistic effect of combined photodynamic chemotherapy on inhibiting tumor growth.	Co-delivery system	[146]
Anti-Glypican-1, oridonin, gadolinium, and Cy7 dye	In vitro In vivo	Pancreatic cancer	Long-time stability and fluorescent/MRI properties; Significant inhibition of viability and apoptosis enhancement; Enabled multimodal targeted imaging.	Theranostic platform for simultaneous diagnosis and effective treatment	[147]
Hydrophobic rapamycin and hydrophilic herbal drug, berberine	In vitro In vivo	Lung cancer	Enhanced internalization and cytotoxicity; Anticancer efficacy; Decreased lung weight and reduction in both number and diameters of lung adenomatous foci and angiogenic markers.	Drug delivery Inhalable nanocomposites	[148]
Gambogic acid and Doxorubicin	In vitro In vivo	Tongue squamous cell carcinoma	Gradual release of DOX and GA under different tumor-specific physiological conditions (low pH and rich HAase); Tumor growth inhibition and significantly prolonged survival rate.	Drug delivery system	[149]
Mn3O4–Ce6	In vitro In vivo	Breast cancer	Homogeneously distributed in whole tumor and significantly reduced the level of intracellular GSH; Intracellular ROS production; Induction of cell death; Complete inhibition of tumor growth.	Sustainable ROS Generator	[150]
Doxorubicin	In vitro (3D) In vivo	Lung cancer	Higher cellular accumulation efficiency and antiproliferation potentials; Superior tumor penetration capability, ROS production level, and cancer cell-killing capacity; Higher tumor accumulation efficiency.	Drug delivery system	[151]
Platinum	In vitro	Lung cancer	Inhibited proliferation, migration and invasion, and induced apoptosis in comparison with cisplatin and carboplatin.	Drug delivery system	[152]
Docetaxel	In vitro	Glioblastoma	Multi-target capability and stronger penetration ability into 3D tumor spheroids’ core; Migrated efficiently across the BBB.	Drug delivery system	[153]
Epigallocatechin-3-gallate and Docetaxel	In vitro In vivo	Prostate cancer	Inhibition of cell growth via induced G2/M phase cell cycle arrest; Significantly attenuated tumor growth and increased M30 protein expression without causing organ damage.	Co-delivery system	[154]
MoS2 quantum dotsCe6	In vitro In vivo	Breast cancer	Appropriate particle size can not only degrade and excrete in a reasonable period induced by redox responsiveness of glutathione but also exhibits a high tumor uptake due to the longer blood circulation time.	Delivery system	[155]
Ultra-small gadolinium oxide	In vitro In vivo	Breast cancer	Rapidly degraded and excreted after reacting with glutathione (GSH) by the redox response; high tumor uptake.	Multimodal imaging; photothermal/radio therapy	[156]
Ultra-small gadolinium oxide and aluminum phthalocyanine	In vitro In vivo	Breast cancer	Enhanced tumor uptake effect; photothermal effect.	Polymer-based multifunctional theranostic/fluorescence/magnetic resonance/photoacoustic imaging	[157]
Chlorin e6 (Ce6)	In vitro In vivo	Cervical cancer	High colloid stability, good biocompatibility, and suitable transverse relaxation rate; High photothermal conversion efficiency and excellent ROS generation efficiency under NIR light irradiation; Significantly high tumor growth inhibition.	Multifunctional nanotheranostic agent Photodynamic/photothermal combined therapy	[158]
Palladium	In vivo	Melanoma	Efficient targeting and effective therapy for CD44-positive tumors such as melanoma.	Drug delivery system	[159]
Disulfiram	In vitro In vivo	Breast cancer	Induces strong cytotoxicity; Passively accumulates in tumors and elicits potent tumor growth inhibition.	Drug delivery system	[160]
Doxorubicin	In vitro	Cervical cancer	Good stability in vitro; Drug release mediated by pH gradient; Lower cytotoxicity in normal cells and higher inhibition ratio in tumor cells; Efficient internalization.	Drug delivery system	[161]
Ce6	In vitro In vivo	Breast cancer	Good biocompatibility; Inhibition of tumor growth.	Delivery system	[162]
Methotrexate and 10-hydroxycamptothecin	In vitro In vivo	Breast cancer	High drug entrapment efficiency and pH/esterase-controlled release behavior; Significant increase in efficiency of selective internalization; Highly synergetic tumor cell-killing and tumor growth inhibition.	Dual-targeting delivery system	[163]
Azobenzene; ammonium polyamidoamine and carboxylatopillar [5]arene	In vitro In vivo	Colon cancer	Good biocompatibility and CRC treatment capability with negligible side effects.	Delivery system	[164]
Doxorubicin	In vitro In vivo	Squamous cell carcinoma	Favorable biocompatibility; relatively low cytotoxicity; good drug loading capability and strong photoacoustic imaging signals; synergistic chemo-photothermal therapy; better therapeutic effects than chemotherapy alone; accumulates at the tumor sites and achieves complete ablation of tumors.	Multifunctional platform in photoacoustic imaging-guided photothermal chemotherapy	[165]
Mitoxantrone and verapamil	In vitro	Breast cancer	Significant cytotoxicity.	Drug delivery system	[166]
Cisplatin	In vitro In vivo	Lung cancer	Specific tumor-targeting ability and redox-responsive drug release manner; effective antitumor performance along with minor side effects and systemic toxicity.	Drug delivery system	[167]
Granzyme B protein	In vitro In vivo	Glioblastoma and Breast cancer	Induced cell apoptosis; accumulated in the solid tumor through enhanced permeability and retention (EPR) effect; Induced tumor cell apoptosis in vivo.	Delivery system	[168]
Curcumin and IR780	In vitro In vivo	Breast cancer	Uniform size, high drug loading ability and excellent colloidal stability; under the NIR condition, IR780 could be triggered to exhibit both PTT/PDT dual-pattern therapy effects, leading to an enhanced therapy efficiency of Cur with good biocompatibility.	Delivery system	[169]
Gemcitabine and imiquimod	In vitro In vivo	Breast cancer	Anticancer activity; suppressed the volume of tumor; imiquimod potentiates the effect of gemcitabine by activating immune cells to suppress tumors.	Drug delivery system	[170]
Photosensitizer (NIR770) and doxorubicin	In vitro In vivo	Lung cancer	Specifically internalized by tumor cells; preferentially retained in mitochondria; highly efficient photothermal therapy and photodynamic therapy upon NIR irradiation; DOX molecules were mainly accumulated in the nucleus.	Synergistic treatment	[171]
Gossypol, Cu(II) and AQ4N	In vitro In vivo	Prostate cancer	Multiple-tumor-targeting ability; accumulates and significantly releases drugs at the tumor region; High antitumor efficiency with negligible side effects.	Delivery system	[172]
Paclitaxel and IR780	In vitro In vivo	Lung cancer	Combinatorial antitumor effects of paclitaxel and IR780 associated with microtubule destruction and mitochondrial apoptotic pathway.	Drug delivery system	[173]
microRNA-31 and Doxorubicin	In vitro	Cervical and Lung cancer	Promoted intracellular accumulation of drugs via the active transport at tumor site; microRNA-31 directly targeted to mtEF4 to promote cell death; synergistic effects.	Co-delivery system	[174]
Folic acid and Dopamine	In vitro In vivo	Melanoma	Improved blood circulation half-life of the drug and prevented premature intravascular drug leakage from the nanocarrier; efficient tumor penetration has shown potential in improving anticancer efficacy.	Co-delivery system	[175]
R820 and Catalase	In vitro In vivo	Melanoma	Selectively targeted melanoma cells with high expression of CD44, and generated oxygen by catalyzing H_2_O_2_, inhibiting tumor growth significantly.	Nanotechnology-based photodynamic therapy	[176]
MnO2-mSiO2	In vitro In vivo	Breast cancer	Almost total suppression of tumor growth without observable recurrence.	Multifunctional nanotheranostic	[177]
Doxorubicin and IR780	In vitro In vivo	Cervical cancer	Selective tumor targeting; synergistic dual-mode chemo-photodynamic therapy against cancers.	Co-delivery system	[178]
Peptide A20-36 (selectively binds to the Ig-BCR of A20 lymphoma cells)	Ex vivo In vivo	B lymphoma	Targeting specificity and kinetics of the NPs; multimodal imaging contrast agents.	Imaging and theranostic applications	[179]
siRNA	In vitro	laryngeal cancer	Downregulation of genes was confirmed; entrapment efficiency of siRNA of 36.8-61.2; significant inhibition of cell growth and induction of apoptosis.	siRNA delivery system	[180]
Doxorubicin	In vitro	Hepatocellular carcinoma	Exhibited H_2_O_2_-responsive release of about 80% DOX and displayed sevenfold selectivity for killing cancer cells over normal cells.	Drug delivery system	[181]
Doxorubicin	In vitro In vivo	Hepatocellular carcinoma	Cellular uptake demonstrated that this system could bind specifically with cancer cells; excellent therapeutic effect by photothermal-chemotherapy.	Drug delivery system	[182]
Doxorubicin	In vitro In vivo	Lung cancer	Suitable drug loading efficiency, excellent solubility, very low hemolytic effect; induction of apoptosis; DNA intercalation, cell cycle arrest at the S phase, light-induced ROS production; inhibition of tumor growth with good safety.	Drug delivery system	[183]
Zinc phthalocyanine	In vitro In vivo	Breast cancer	Good ability for infrared thermal, photoacoustic, fluorescence, and X-ray computed tomography imaging, high photo-heat conversion efficiency for photothermal therapy; tumor growth inhibition; excellent combined therapeutic effect.	Smart theranostic nanoplatform multimodal imaging-guided combined phototherapy	[184]
Cyclodextrin and amantadine	In vitro	Breast cancer	Excellent fluorescence; internalized into tumor cells via HA receptor CD44-mediated endocytosis; effective targeted tumor cell imaging.	Cancer diagnosis and treatment	[185]
Doxorubicin	In vitro	Lung cancer	Higher cytotoxicity; inhibited tumor cell invasion and metastasis by downregulating N-cadherin expression.	Drug delivery system	[186]
Doxorubicin	In vitro In vivo	Ovarian cancer	High selectivity resulting in strong killing; long elimination half-life, elevated tumor accumulation and effective inhibition of the ovarian tumor.	Drug delivery system	[187]
Doxorubicin and CuS	In vitro In vivo	Breast cancer	Good biocompatibility; targeting effect; synergistic combination of chemo- and phototherapy; potential for tumor diagnosis and treatment.	Drug delivery system	[188]
Mn-modified phthalocyanine derivative and docetaxel	In vitro In vivo	Lung cancer	Activated tumor immunity through cGAS–STING and chemotherapy; effectively inhibited tumor cell growth.	Delivery system	[189]
MicroRNA-34a	In vitro	Lung cancer	Successful delivery and uptake resulted in altered ATP levels, decreased glycolytic flux, Nrf-2, and glutathione levels, ultimately resulting in caspase-3 activation and apoptosis; underlying molecular changes in epigenetic status of D loop on the mtDNA and transcription of mtDNA-encoded genes.	Delivery system	[190]
Chitosan	In vitro	Breast cancer	Low hemolysis; high resistance to bovine serum albumin adsorption; efficient internalization; non-toxic to human skin fibroblasts.	Drug Nanocarrier or drug delivery system	[191]
siRNA	In vitro In vivo	Melanoma	Significant inhibitory effect against melanoma cells; siRNA liposomes may inhibit tumor growth by downregulating surviving; survivin–siRNA cationic liposome nanoparticles were able to effectively inhibit proliferation and migration of melanoma cells in vitro and in vivo, probably by inhibiting survivin–mRNA and protein expression.	siRNA delivery	[192]
MicroRNA-125b	In vitro In vivo	Lung cancer	Increase in M1 to M2 macrophage ratio and 300-fold increase in the iNOS (M1 marker)/Arg-1 (M2 marker) ratio; intraperitoneally administered macrophage-specific NPs can successfully transfect tumor-associated macrophages (TAMs) in lung tissues of both naïve mice and a KP-GEM NSCLC mouse model; successful TAM repolarization toward M1 phenotype has significant implication in anticancer immunotherapy.	Transfection system	[193]
Paclitaxel in combination with MicroRNA-125b	In vitro In vivo	Ovarian cancer	Specifically targets TAMs in the peritoneal cavity and can repolarize macrophages to an immune-activating phenotype; enhances antitumor efficacy of paclitaxel during later stages of disease progression as seen by significant reduction in ascitic fluid and peritoneal VEGF levels; does not induce systemic toxicity.	Delivery system	[194]
miRNA 145	In vitro In vivo	Colon cancer	High up-conversion emission and good monodispersity; Excellent biocompatibility; High level of cellular uptake and miR-145 expression, resulting in significant cell cycle arrest in G1 and inducing CCND1, CDK6, and CCNE2 protein downregulation; inhibition of tumor growth.	Delivery system	[195]
Plasmid DNA	In vitro	Cervical and Lung cancer	Higher transfection efficiency; stable up to a week at 4 degrees.	Delivery and transfection system	[196]
MTH1 inhibitor–TH287 and MDR1 siRNA	In vitro	Oral cancer	Effective in controlling drug release and internalization; reduced tumor burden; inhibited MDR1 function and enhanced cell-killing effect.	Delivery system	[197]
Cyanine 3 (Cy3)-labeled siRNA	In vitro In vivo	Lung cancer	Effectively delivered Cy3-labeled siRNA to cancer cells via receptor CD44 and inhibited cell proliferation by BCL2 downregulation; Inhibition of tumor growth by BCL2 downregulation.	Delivery system	[198]
Anti-miR21 and Resveratrol		Gastric carcinoma	Higher cellular internalization; anticancer effect of the optimized formulation and synergistic effects of anti-miR21 and RSV; induction of apoptosis and cell necrosis.	Delivery system	[199]
Paclitaxel	In vitro In vivo	Lung cancer	Antitumor growth activity.	Nanocarrier	[200]
Paclitaxel	In vitro	Ovarian cancer	Selectively targeted and entered CD44-overexpressing cancer cells via receptor-mediated endocytosis.	Drug delivery system	[201]
siRNA Doxorubicin	In vitro	Ovarian Cancer Colorectal Cancer	Formation of stable complexes with siRNA; prevented RNase-mediated siRNA degradation; increased cancer cell specificity and enhanced cytotoxic effect in CD44+ cells.	Co-delivery system	[202]
Doxorubicin (DOX) and photosensitizer chlorin e6 (Ce6)	In vitro In vivo	Melanoma	Higher cellular uptake and remarkably better tumor-targeted accumulation than free drugs; with laser irradiation, anticancer activities were enhanced both in vitro and in vivo.	Chemo-photodynamic therapy	[203]
Dexamethasone and Doxorubicin	In vitro Ex vivo	Breast cancer Colorectal Cancer Human whole blood	DEX suppressed cytotoxicity of DOX; synergistically enhanced cytotoxicity;in an ex vivo human whole blood sample, found activation of complement and coagulation cascade in one group of donors. Encapsulation of DOX within the nanoparticle core eliminated such deleterious side effects.	Drug delivery system	[204]
Doxorubicin	In vitro In vivo	Breast cancer Colon cancer	High targeting and antitumor activity against CD44 receptors; longer circulation time and higher accumulation in 4T1 tumors.	Drug delivery system	[205]
Oleic acid	In vitro	Breast Cancer Melanoma	Efficient delivery of oleic acid; greater uptake by cancer cells (expressing CD44 receptors) than normal cells.	Drug delivery system	[206]
Paclitaxel	In vitro In vivo	Lung cancer	Greater in vitro cytotoxicity and apoptosis; much higher antitumor efficacy and improved safety profile.	Drug delivery system	[207]
anti-Gasdermin B antibody	In vitro In vivo	Breast cancer	Reduces diverse protumor functions (migration, metastasis, and resistance to therapy)	Delivery system	[208]
5-Fluorouracil	In vitro In vivo	Skin cancer	Non-irritant; permeability properties; cytotoxic effect; favorable biosafety; good antitumor effects.	Topical gel for drug delivery	[209]
Doxorubicin	In vitro	Hepatocellular carcinoma	Effectively avoids biological barriers; provides long blood circulation and achieves high tumor accumulation; fast elimination from tumor and released the loaded drugs for chemotherapy after UV-induced dissociation; good targetability to CD44 receptors.	Drug delivery system	[210]

## Data Availability

Not applicable.
